# Early-age feed restriction affects viability and gene expression of satellite cells isolated from the gastrocnemius muscle of broiler chicks

**DOI:** 10.1186/2049-1891-3-33

**Published:** 2012-11-06

**Authors:** Yue Li, Xiaojing Yang, Yingdong Ni, Eddy Decuypere, Johan Buyse, Nadia Everaert, Roland Grossmann, Ruqian Zhao

**Affiliations:** 1 Key Laboratory of Animal Physiology & Biochemistry, Nanjing Agricultural University, Nanjing 210095, China; 2 Laboratory of Livestock Physiology, Immunology and Genetics, K.U. Leuven, Leuven, Belgium; 3 Department of Functional Genomics & Bioregulation, Institute of Animal Genetics Mariensee, FLI, 31535, Neustadt, Germany

**Keywords:** Feed restriction, Satellite cells, Proliferation, Gene expression, Chicken

## Abstract

**Background:**

Muscle growth depends on the fusion of proliferate satellite cells to existing myofibers. We reported previously that 0–14 day intermittent feeding led to persistent retardation in myofiber hypertrophy. However, how satellite cells respond to such nutritional insult has not been adequately elucidated.

**Results:**

One-day-old broiler chicks were allocated to control (Con, *ad libitum* feeding), intermittent feeding (IF, feed provided on alternate days) and re-feeding (RF, 2 days *ad libitum* feeding after 12 days of intermittent feeding) groups. Chickens were killed on Day 15 and satellite cells were isolated. When cultured, satellite cells from the IF group demonstrated significant retardation in proliferation and differentiation potential, while RF partly restored the proliferation rate and differentiation potential of the satellite cells. Significant up-regulation of insulin like growth factor I receptor (IGF-IR) (*P<*0.05) and thyroid hormone receptor α (TRα) (*P<*0.05), and down-regulation of growth hormone receptor (GHR) (*P<*0.01) and IGF-I (*P<*0.01) mRNA expression was observed in freshly isolated IF satellite cells when compared with Con cells. In RF cells, the mRNA expression of IGF-I was higher (*P<*0.05) and of TRα was lower (*P<*0.01) than in IF cells, suggesting that RF restored the mRNA expression of TRα and IGF-I, but not of GHR and IGF-IR. The Bax/Bcl-2 ratio tended to increase in the IF group, which was reversed in the RF group (*P<*0.05), indicating that RF reduced the pro-apoptotic influence of IF. Moreover, no significant effect of T_3_ was detected on cell survival in IF cells compared with Con (*P*<0.001) or RF (*P<*0.05) cells.

**Conclusions:**

These data suggest that early-age feed restriction inhibits the proliferation and differentiation of satellite cells, induces changes in mRNA expression of the GH/IGF-I and thyroid hormone receptors in satellite cells, as well as blunted sensitivity of satellite cells to T_3_, and that RF partially reverses these effects. Thus, a moderate nutritional strategy for feed restriction should be chosen in early chick rearing systems.

## Background

Skeletal muscle accounts for 40–50% of body weight, and is the most important product for the poultry industry. Nutritional and metabolic exposure during critical periods of early development can have a long-term programming effect on health in adulthood [1]. This “nutritional or metabolic programming” has been described not only in mammals [2-4], but also in avian species. A large number of studies have been done in chickens to investigate the long-term effect of early nutritional manipulation on body and muscle growth [3,5]. Intermittent feeding, chickens fed *ad libitum* on one day and fasted on the other day, is one of the common strategies of feed restriction for higher feeding efficiency in the poultry industry in China. However, we found this kind of “feed restriction” could have a long-term negative influence in skeletal muscle growth if endured during the early growth period (0–14d) [5].

As a source of nuclei for muscle growth, satellite cells must also be affected. Skeletal muscle growth in post-hatch birds is dictated by the accumulation of nuclei in muscle fibers and hypertrophy, the terminally differentiated myofibers are incapable of mitosis for myofiber accretion. Satellite cells are capable of entering the cell cycle to proliferate, differentiate and contribute nuclei to existing myofibers [6,7]. In broilers, satellite cells demonstrate high activity in proliferation and differentiation 1 week post-hatch. After this, the satellite cell population declines dramatically [8,9], although satellite cells retain mitotic activity as nuclei donors for myofiber hypertrophy. Therefore, the proliferative potential of satellite cells during the early post-hatch period would affect muscle hypertrophy in later life. The proliferative potential of satellite cells is highly sensitive to nutritional state [10]. It has been shown that early post-hatch starvation decreases satellite cell proliferation and skeletal muscle growth in chicks [8] and turkeys [10-12], which can be restored by re-feeding. Most previous studies focus on the first 2 or 3 days post-hatching, and only on the immediate effect after feed restriction. However, what happens during prolonged feed restriction such as 1 or 2 weeks after hatching and what are the differences between the immediate effects after fasting and after re-feeding, and how does satellite cell proliferation and differentiation respond to these situations. Moreover, proliferation and apoptosis (programmed cell death) together comprise normal cell growth regulation. Only the viability of the number of living cells (MTT assay) could reflect the ultimate balance between cell proliferation and apoptosis. The MTT assay measures cell survival and proliferation, although the classic methods for cell proliferation studies are more sensitive and accurate compared with the MTT assay.

Endocrine factors have been shown to be involved in the regulation of satellite cell proliferation [13-15]. Satellite cell proliferation was decreased in early post-hatch starved chicks paralleling lower growth hormone receptor gene expression [8]. The activity of satellite cells was decreased during the first week of underfeeding in young sheep, which coincided with reduced muscle insulin like growth factor I (IGF-I) mRNA levels. In contrast, IGF-I gene expression was increased during long-term underfeeding causing muscle necrosis, suggesting activation of satellite cells for muscle repair [16]. IGF-I also induced DNA synthesis in avian skeletal muscle satellite cells *in vitro*[14]. This information suggests a role of growth hormone (GH)/IGF-I in mediating the effects of under-nutrition on satellite cell proliferation. How satellite cell GH receptor and IGF-I receptor expression responds to long-term early-age feed restriction has not been adequately elucidated.

Thyroid hormone (T_3_,T_4_) levels also reflect nutritional state and regulate cell proliferation in a dose-dependent manner [15]. Satellite cells isolated from muscles of hypothyroid rats are less active in proliferation and differentiation at the start of culture [17]. We reported previously that intermittent (skip a day) feeding the first 2 weeks after hatching caused a persistent decrease in serum levels of T_3_[5]. However, little is known whether long-term early post-hatch underfeeding affects TRα mRNA expression in satellite cells or whether satellite cell responsiveness to T_3_*in vitro* is influenced.

Therefore, we have used satellite cells isolated from muscle of chickens subjected to nutritional intervention to investigate the impact of early-age feed restriction and re-feeding on proliferation/differentiation potentials, mRNA expression of relevant genes, as well as the responsiveness of satellite cells to T_3_.

## Materials and methods

### Animals and experimental design

One-day-old San Huang chicks (a crossbred local broiler breed) were allocated randomly to the control (Con, fed *ad libitum*), intermittent feeding (IF, fed *ad libitum* on alternate days for 14 days) and re-feeding (RF, 2 days *ad libitum* feeding after 12 days of intermittent feeding) groups (N = 10/group). The diets were formulated according to the nutritional requirements of the breed and all the chicks were raised under standard conditions recommended by the breeding company. Chickens were killed on Day 15 (feeding day for all 3 groups) and satellite cells were isolated from the lateral gastrocnemius muscle [18] for RNA extraction and cell culture immediately. The experiment was repeated 3 times following the guidelines of the regional animal ethics committee.

### Cell culture

Satellite cells were isolated from the lateral gastrocnemius muscles according to a protocol described by Doumit and Merkel [18] with some modifications. Briefly, cells were dissociated by digestion with Pronase (1 g/L, Roche, Switzerland) and purified (to remove fibroblasts or other types of cells) by using Percoll (Sigma-Aldrich, Germany) gradient centrifugation [19,20]. The isolated satellite cells were verified by Desmin antibody immunostaining (up to 98% desmin positive). The satellite cells from 10 chickens pooled as one sample for cell culture. A total of 30 chickens for each group were used, and the samples for cell culture were 3 per group.

For proliferation/differentiation analysis, satellite cells from 3 different groups were plated immediately after Percoll purification at 5×10^4^ cells/cm^2^ in DMEM supplemented with 10% horse serum and 10% fetal bovine serum (FBS) [21] (standard serum-rich medium), and maintained at 37°C in a humidified incubator containing 95% air and 5% CO_2_. Cell viability was assessed by the MTT (Sigma-Aldrich, Germany) assay [22]. Briefly, cells were seeded at 10^4^ cells per well in a 96-well plate and incubated in 200 μL medium for 1, 2, or 3 days, 6 wells for each group and each day. This was followed by adding 25 μL of the MTT solution (5 mg/mL; Sigma-Aldrich, Germany), while cells were protected from light. After 4 h incubation, under standard conditions of 5% CO_2_ and 37°C, the purple formazan product became visible. The precipitated formazan was dissolved by adding 100 μL dimethyl sulfoxide (DMSO) and placing it on a shaker for 5 minutes. An increase in cell number results in an increase in the amount of MTT formazan formed and an increase in absorbance. The absorbance was read on ELISA plate reader at 570 nm. The blank values (medium) were subtracted from each well of the untreated and treated cells. Morphological changes of cultured cells were also observed and photographed for 3 days to reflect cell proliferation and differentiation capacity.

For the T_3_ treatment, cells from the different groups were dispersed immediately after Percoll purification with DMEM supplemented with 10% horse serum, 10% FBS, plated in 96-well plates at an initial density of 10^4^ cells per well, and allowed to attach overnight. Then cells were rendered quiescent in DMEM supplemented with 1% FBS (low serum medium) for 24 h [23,24]. Satellite cells from the different groups were treated and grown with or without 2×10^-8^ M 3^′^,3^′^,5^′^-triiodo-L-thyronine (T_3_, Sigma-Aldrich, Germany) in basal medium (low serum medium) (according to the literature and our preliminary experiment) [25]. Cell viability was measured by the MTT assay at 24 h after T_3_ treatment.

### RNA extraction, reverse transcription (RT) and real-time PCR

Total RNA of satellite cells was extracted, quality verified and 2 μg of total RNA was reverse transcribed as described previously [5]. Real-time PCR was performed to quantify the mRNA expression of GHR, IGF-I, IGF-IR, TRα, Bax and Bcl-2 with the Mx3000P Real-Time PCR System (Stratagene, USA). mRNA expression of target genes were quantified relative to β-actin. The nucleotide sequences of the primers and the PCR conditions for these genes were shown in Table 1. The method of 2^-ΔΔCt^ was used to analyze the data of real-time PCR [26]. Briefly, according to the formula *ΔΔ*Ct = (Ct. _Target_–Ct. _*β* − actin_)_sample_–(Ct. _Target_–Ct. _*β* − actin_)_mean of control_, every sample had data from 2^-ΔΔCt^.

**Table 1:** Nucleotide sequences of specific primers and real-time PCR conditions for satellite cells

**Target genes**	**GenBank accession no.**	**Primer sequences**
β-actin	GenBank NM205518	F: 5^′^- tgcgtgacatcaaggagaag −3^′^
		R: 5^′^- tgccagggtacattgtggta −3^′^
GH-R	GenBank NM_001001293	F: 5^′^- aacgaggacacttacttcaccaca −3^′^
		R: 5^′^- gcatttccatacttggggtttct −3^′^
IGF-IR	GenBank AJ223164	F: 5^′^- gcagaggagagtgaggtggaa −3^′^
		R: 5^′^- gtaaaaggctggagatgggaga −3^′^
IGF-I	GenBank M32791	F: 5^′^- tgtgctccaataaagccacct −3^′^
		R:5^′^- tttctgtttcctgtgttccctctac −3^′^
TRα	GenBank NM_205313	F: 5^′^-tctgcgtggataagatagagaagtg-3^′^
		R: 5^′^- gttgtgtttgcggtagttgatgtag −3^′^
Bcl-2	GenBank D11381	F: 5^′^- gccccccgcctcaccatg −3^′^
		R: 5^′^- cccggggtgagccatggtttc −3^′^
Bax	GenBank NM_007527	F: 5^′^- acagggtttcatccaggatcgagca-3^′^
		R: 5^′^- tcagcttcttggtggacgcatc −3^′^

Real-time PCR was programmed as follows: initial denaturation at 95°C for 10 min, followed by 40 cycles of denaturation at 94°C for 20 s, annealing at 62°C for 30 s to all the genes except 64°C for 30 s to IGF-IR, and extension and data collection at 72°C for 30 s.

### Statistical analysis

The results were expressed as the Mean ± SEM. All data were subjected to one-way ANOVA analysis testing the main effect of the treatment. When the main effect of treatment was significant, statistical differences of the means were assessed by least-significant difference. *P*<0.05 was considered significant. All statistical analyses were performed with SPSS11.0 for Windows (StatSoft Inc., USA).

## Results

### Proliferation and differentiation of satellite cells

**Figure 1:**
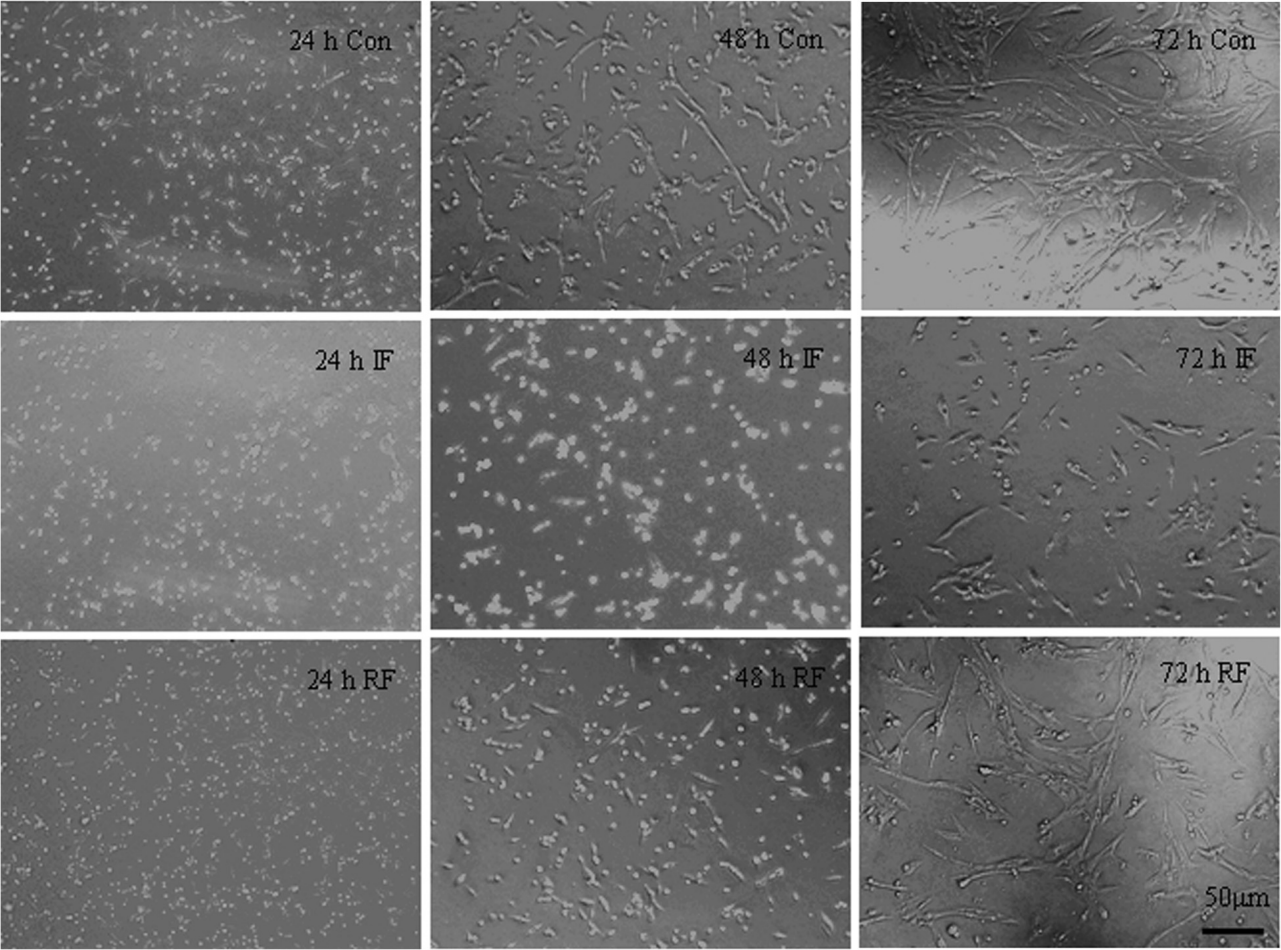
**Morphologic development of the satellite cells in culture according to feeding group at 15 days of age.** Optical phase contrast images in Con (control group), IF (intermittent feeding group) and RF (re-feeding group), bar: 50 μm. Cells were seeded at 5×10^4^ cells/cm^2^ in standard serum-rich medium in 6-well plates (34.8-mm petri dishes). Morphological changes were followed during the first 3 days to reflect cell proliferation and differentiation capacity.

**Figure 2:**
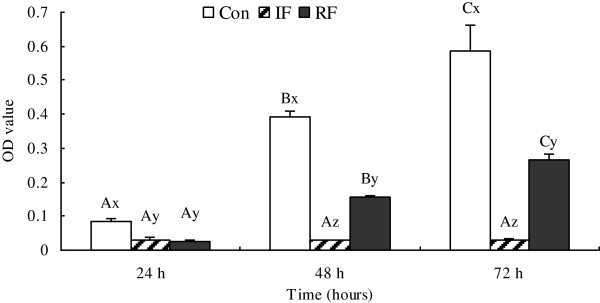
**Viability of satellite cells according to feeding group at 15 days of age.** Con: control group; IF: intermittent feeding group; RF: re-feeding group. Cells were seeded at 10^4^ cells per well in a 96-well plate, 6 wells for each group and each day. Cell viability was assessed by the MTT assay as OD value. The absorbance was read on ELISA plate reader at 570 nm. Values are means ± SEM. Groups not sharing a common letter within the same figure are significantly different (A–C: significance within the same group; x–z: significance within the same time). *P*<*0.05*, N = 6/group/day, N is the repeat number in cell culture for one feeding group in one day per time, the experiment was repeated three times.

As shown in Figure 1, the morphologic development of the satellite cells was obviously different among the three treatment groups. Satellite cells from the Con group were beginning to align 24 h after plating, myotubes were formed in 48 h, and differentiation almost completed by 72 h. Cells from the IF group demonstrated significant retardation in myotube differentiation which started 72 h after seeding, 2 days later than the control group. In RF cultures, myotubes started to form at 48 h. In agreement with the morphological observations, cell viability, as shown in MTT values, demonstrated the same pattern at 24, 48 and 72 h of culture (Figure 2). Cells from the Con group showed the highest viability at each time points (*P*<0.001), whereas the cell viability of the IF group was significantly depressed at all the 3 time points tested. The MTT values were partly restored in the RF group, but were less than 50% of the values in the Con group at 48 and 72 h. The MTT result was significantly increased compared with the previous day in the Con group, the OD value on 48 h was 4.5 fold higher than on 24 h, and the OD value on 72 h was 0.5 fold higher than on 48 h (*P*<0.001). Proliferation in the IF group was arrested and no significant changes were observed in cell viability at 48 or 72 h. RF treatment restored cell viability, the OD value on 48 h was about 6.5 fold higher than on 24 h, and the OD value on 72 h was 0.5 fold higher than on 48 h (*P*<0.001).

### Abundance of relevant gene transcripts in satellite cells

**Figure 3:**
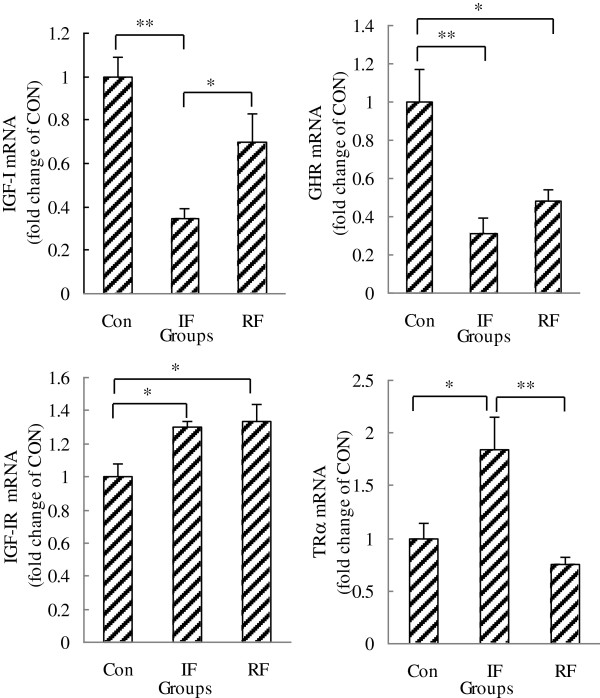
**IGF-I, GHR, IGF-IR and TRα mRNA gene expression of satellite cells according to feeding group at 15 days of age.** Con: control group; IF: intermittent feeding group; RF: re-feeding group. One RNA sample of satellite cells was extracted after satellite cells isolated from the muscle of 10 chickens from one group immediately after death on Day 15. Values are means ± SEM. Significant differences are indicated (*P<0.05, **P<0.01). N = 3/group, N is the number of samples for cell culture. The satellite cells from 10 chickens pooled as one sample for cell culture.

As shown in Figure 3, GHR and IGF-I mRNA levels were down-regulated (*P*=0.005, *P*=0.003, respectively) while IGF-IR and TRα mRNA expression was up-regulated (*P*=0.035, *P*=0.023, respectively) by intermittent feeding compared with the Con group. RF resisted this down-regulation on IGF-I and up-regulation on TRα caused by under-nutrition. The mRNA expression of TRα in the RF group was lower than that in the IF group (*P*=0.008), the mRNA expression of IGF-I was higher than that in the IF group (*P*=0.036), and these gene transcripts were not significantly different with those in the Con group. However, the GHR mRNA level was still lower (*P*=0.017) and the IGF-IR mRNA level was still higher (*P*=0.022) in the RF group compared with the Con group.

**Figure 4:**
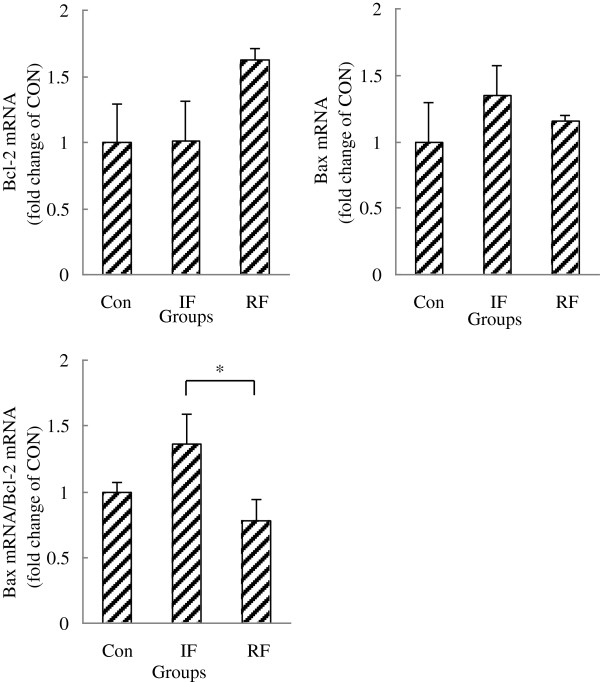
**Bax, Bcl-2, Bax mRNA/Bcl-2 mRNA gene expression of satellite cells according to feeding group at 15 days of age.** Con: control group; IF: intermittent feeding group; RF: re-feeding group. One RNA sample of satellite cells was extracted after satellite cells isolated from the muscle of 10 chickens from one group immediately after death on Day 15. Values are means ± SEM. Significant difference is indicated (*P<0.05). N = 3, N is the culture numbers.

To show whether early-age feed restriction and re-feeding affect apoptotic potential of satellite cells, the mRNA abundance of a pro-apoptotic gene (Bax) and an anti-apoptotic gene (Bcl-2) were measured. No differences in Bax and Bcl-2 mRNA expression were observed among the 3 treatment groups, while Bax/Bcl-2 ratio, which indicates susceptibility to apoptosis, was decreased by re-feeding compared with the IF group (Figure 4, *P*=0.045).

### Responsiveness of satellite cells to T_3_

**Figure 5:**
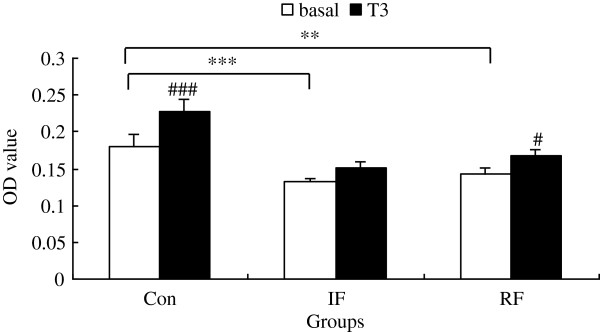
**Effect of T**_**3**_**on viability of chicken satellite cells at 24 hours according to feeding group.** Con: control group; IF: intermittent feeding group; RF: re-feeding group. Cells were seeded at 10^4^ cells per well in a 96-well plate, 6 wells for each group and allowed to attach overnight. Then cells were rendered quiescent in low serum medium for 24 hours. Three wells of each group were treated with or without T_3_ in low serum medium for another 24 hours. Cell viability was assessed by the MTT assay as OD value. Values are means ± SEM. Significant differences are indicated, **–***: significance within the basal medium among three different feeding strategy groups (**P<0.01, ***P<0.001), #–###: significance within the same feeding strategy group after T_3_ treatment vs. basal (#: *P*<0.01, ###: *P*<0.001). N = 3, N is the culture numbers.

As shown in Figure 5, the differences in cell viability among the 3 groups exhibited similar patterns as shown in Figure 2, although the differences under low serum medium were not as pronounced as that under standard serum-rich medium. Cell viability was decreased in the IF group and the RF group compared with the Con group (*P*<0.001, *P*=0.002, respectively). Cells from the Con group responded to T_3_ with significantly increased cell viability (*P*<0.001), while cells from the IF group were insensitive to T_3_. RF restored the sensitivity of satellite cells to T_3_ treatment (*P*=0.03).

## Discussion

Previous studies regarding the nutritional influences on satellite cells have been focused on the changes of cell mitotic activity by determining DNA synthesis with incorporation of either ^3^H]thymidine [8,10] or BrdU [8,11,12,27]. Pax7 is also used as a specific marker for satellite cells in immunohistochemistry [27]. These are classic methods for cell proliferation studies, which are more sensitive and accurate compared with the MTT assay. However, normal cell growth regulation not only includes proliferation but also apoptosis (programmed cell death). The MTT assay is a convenient and efficient method for establishing the number of living cells (viability), reflecting the ultimate balance between cell proliferation and apoptosis. Here, the morphological differences of the satellite cells revealed the significant impact of early-age intermittent feeding on cell proliferation and differentiation, while the changes in the ratio of Bax to Bcl-2 mRNA expression implicated changes of apoptotic potential of the satellite cells responding to nutrition restriction and restoration. This implies some strategies of feed restriction for higher feeding efficiency should be appropriately (for example, fasted one day per three days) and duly (for example, fasted at an older age). If not, the total number of satellite cells will be less for full muscle growth.

We found that 2 days re-feeding after 12 days of intermittent feeding was unable to restore completely the proliferation and differentiation capabilities of the satellite cells, as indicated by the cell morphology and viability detected with the MTT assay. This result adds to the previous findings that several days of re-feeding was able to completely reverse the depressed mitotic activity of the satellite cell caused by short-term (2–3 days) feed deprivation or fasting in chicks or turkeys [10,12,27,28]. A delayed peak of satellite cell DNA synthesis and mitotic activity was observed after 2–3 days of re-feeding in chicks [8] and 3 days re-feeding in young turkeys [27], which allows a complete restoration of satellite cell numbers. Difference in fasting strategies should take into account this divergence. For those starter diet withdrawal treatments, the fasting only lasted 2 or 3 days after hatching, when yolk residue still serves as an energy resource. It is possible that intermittent (skip a day) feeding for 2 weeks after hatching is more stringent compared with the short-term fasting in other studies, 2 days of re-feeding was not sufficient for restoring satellite cell numbers. Another possibility is the critical window during early post-hatch development for satellite cell proliferation. The first week after hatching is considered as the most active period for satellite cell proliferation and differentiation. Re-feeding occurring within this period may be more effective for a complete compensation, compared with the delayed re-feeding in this study. The most dramatic change in satellite cell activity may occur within the first week, yet the decreased satellite cell activity observed on Day 15 in this study reflects the cumulative effects of intermittent feeding in the first 2 weeks of post-hatch life. This also implicates a suited period for feed restriction and re-feeding that should be considered.

Previous findings have shown a role for apoptosis in muscle induced by under-nutrition [28-30], so here we also tested the expressions of apoptotic regulatory factors, Bax, a death-promoting molecule, and Bcl-2, a survival protein, in extracted satellite cells to explore the survival of them in different feed treatments [31,32]. We noticed in our previous research that early feed restriction decreased the mRNA expression of Bcl-2 and increased the ratio of Bax mRNA/Bcl-2 mRNA in gastrocnemius muscle tissue at the end of 14 days of early-age feed restriction, but there was no difference in the evaluation of DNA ladder electrophoresis (data not published). However, no changes were found here in the mRNA levels of Bcl-2 and Bax in satellite cells of the three feed treatment groups, and there was no difference in Bax/Bcl-2 ratio between the RF and Con groups. It may be that the 14 days of alternate fasting did not induce apoptosis obviously or exhibited in these factors. We found a down-regulation of Bax/Bcl-2 ratio in the RF group compared with the IF group, suggesting satellite cell apoptosis was repressed by restoration of nutrition during re-feeding.

It is suggested that the GH/IGF-I system mediates the effect of nutritional state on satellite cells [33]. Feed restriction induces a significant fall in circulating IGF-I [34,35] and a rise in plasma GH [36], which could be restored to the normal levels by re-feeding [37]. We reported previously that IF chickens expressed lower IGF-I and higher IGF-IR mRNA in the gastrocnemius muscle on Day 14 [5]. Satellite cells isolated from the muscle showed similar responses with lower GHR, IGF-1 and higher IGF-IR mRNA expression in the IF group. It was suggested that in chickens after hatching, hepatic gene expression of IGF-I is GH-dependent while muscular gene expression of IGF-I is independent of GH and GHR [38]. However, it is unknown whether IGF-I expression in satellite cells is dependent on GHR. Here, expression of GHR and IGF-I in satellite cells exhibited a similar pattern in response to feed restriction and re-feeding, suggesting a possible regulatory link between these two genes.

The role of the GH/IGF-I axis in the regulation of avian muscle growth remains obscure [39]. Growth hormone can promote skeletal muscle satellite cell proliferation *in vitro*[13,14] and *in vivo*[40,41], and modify GHR expression [13,14]. Satellite cell proliferation was decreased in starved chicks along with a lower GHR gene expression, which were reversed with re-feeding [8]. IGF-I stimulates the proliferation [14], and fusion of satellite cells *in vitro*[42-46]. However, IGF-I together with GH in culture showed no enhancement effect on DNA synthesis in chicken satellite cells [47]. Since both myofibers and satellite cells are able to produce IGF-I, the effects of paracrine and autocrine IGF-I on satellite cell activity have to be considered, in addition to the role of endocrine IGF-I. Recently, mechano growth factor E (MGF-E), derived from an isoform of IGF-I, was reported to activate human muscle progenitor cells [48].

In addition to the GH/IGF system, thyroid hormones were suggested to be involved in mediating the effect of nutrition on satellite cell function [15,49]. Subcutaneous injections of T_4_ in rats would stimulate the number of total satellite cells and satellite cells per muscle fiber [50], while satellite cell numbers extracted from the hypothyroid rats were fewer and less active in proliferation and differentiation at the start of culture [17]. However, it is unclear how expression of the thyroid hormone receptor in satellite cells responds to nutritional status and thyroid hormone levels. We reported previously that serum concentrations of both T_3_ and T_4_ decreased with IF for 14 days in chicks [5]. We observed a significant up-regulation of TRα mRNA expression in the IF group, which was completely restored with re-feeding. This up-regulation of TRα mRNA expression in satellite cells may represent a feedback regulation through decreased serum thyroid hormone levels. However, the TRα mRNA expression in satellite cells was not coinciding with the viability of satellite cells (Figures 2 and 3). It is speculated that the thyroid hormone receptor activity, which determines the sensitivity of the satellite cells to T_3_, may be blunted. This speculation was supported in the T_3_ challenge test for satellite cells from the three different groups (Figure 5). Satellite cells from the IF group were insensitive to T_3_ while re-feeding partly restored the responsiveness of satellite cells to T_3_, although the viabilities were still significantly lower compared with the Con group at both basal and T_3_-stimulated conditions. It is likely that the up-regulation of TRα mRNA expression in the IF group represents a feedback mechanism of disrupted signaling of thyroid hormones on satellite cells.

In conclusion, long-term feed restriction (12–14 days of intermittent feeding) immediately after hatching impairs proliferation and differentiation capabilities of satellite cells, which could not be completely restored by 2 days of re-feeding. The disrupted satellite cell viability was associated with alterations in mRNA expression of the GH, IGF-I and thyroid hormone receptors, as well as the blunted sensitivity of satellite cells to T_3_. Therefore, the persistent retardation in myofiber hypertrophy caused by 14 days of intermittent feeding post-hatching reported previously [5] can be explained by the decreased satellite cell proliferation and differentiation activity, lower serum T_3_ levels and the blunted sensitivity of satellite cells to T_3_. This suggests that long-term IF carried too early after hatching is not an ideal strategy for poultry meat production. RF partially reverses these effects, which indicates a moderate nutritional strategy for feed restriction if implemented early post-hatching.

## Competing interests

The authors declare that they have no competing interests.

## Authors’ contributions

YL carried out the experiments, participated in the data collection, data analysis and interpretation, and drafted the manuscript. XY and YN helped in data analysis and interpretation, and paper drafting. ED, JB, NE and RG provided valuable advice for this study and helped in editing the manuscript. RZ contributed in conception, experimental design, data interpretation and finalized the manuscript. All authors read and approved the final manuscript.
